# On the role of microkinetic network structure in the interplay between oxygen evolution reaction and catalyst dissolution

**DOI:** 10.1038/s41598-020-69723-3

**Published:** 2020-08-24

**Authors:** An Phuc Dam, Georgios Papakonstantinou, Kai Sundmacher

**Affiliations:** 1 Department Process Systems Engineering, Max Planck Institute for Dynamics of Complex Technical Systems, Sandtorstr.1, 39106 Magdeburg, Germany; 2 Department of Process Systems Engineering, Otto-Von-Guericke University Magdeburg, Universitätsplatz 2, 39106 Magdeburg, Germany

**Keywords:** Corrosion, Electrocatalysis, Corrosion, Electrocatalysis, Electrochemistry, Corrosion, Electrocatalysis, Hydrogen energy, Hydrogen storage

## Abstract

Understanding the pathways of oxygen evolution reaction (OER) and the mechanisms of catalyst degradation is of essential importance for developing efficient and stable OER catalysts. Experimentally, a close coupling between OER and catalyst dissolution on metal oxides is reported. In this work, it is analysed how the microkinetic network structure of a generic electrocatalytic cycle, in which a common intermediate causes catalyst dissolution, governs the interplay between electrocatalytic activity and stability. Model discrimination is possible based on the analysis of incorporated microkinetic network structures and the comparison to experimental data. The derived concept is used to analyse the coupling of OER and catalyst dissolution on rutile and reactively sputtered Iridium oxides. For rutile Iridium oxide, the characteristic activity and stability behaviour can be well described by a mono-nuclear, adsorbate evolution mechanism and the chemical type of both competing dissolution and rate-determining OER-step. For the reactively sputtered Iridium oxide surface, experimentally observed characteristics can be captured by the assumption of an additional path via a low oxidation state intermediate, which explains the observed characteristic increase in OER over dissolution selectivity with potential by the competition between electrochemical re-oxidation and chemical dissolution.

## Introduction

PEM water electrolysis is considered as one of the most promising power-to-X technologies to store energy from intermittent, renewable power sources. Due to the acidic environment of oxygen evolution reaction (OER) in PEM water electrolysers, electrocatalysts need to be highly stable against corrosion. Since Iridium and Ruthenium are characterised by both high activity and stability^1^, they are widely considered as possible base materials for the synthesis of OER catalysts. However, due to the high scarcity of these metals, catalyst loading must be decreased significantly to allow wider application within the future energy system. At low loadings, catalyst corrosion becomes a major challenge, also for Iridium or Ruthenium based metal oxide catalysts. In a passivated state, most noble metals, except the least stable palladium, do not show significant dissolution at steady-state conditions below onset potential of OER^2,3^. Without prior reduction step, Iridium and Ruthenium show no significant dissolution below OER onset potential, even under highly dynamic polarisation^3^. However, above the onset potential for oxygen evolution, catalyst dissolution is observed also for noble metals under both dynamic and steady-state conditions^2–5^.

While metallic surfaces show a transient behaviour under potentiostatic polarisation^7^, for many metal oxide surfaces a quasi-steady-state behaviour can be observed in the OER region^2,6^. For various noble metal oxide surfaces as well as some Iridium-based perovskites, a constant ratio between oxygen evolution and catalyst dissolution rates has been reported, at different conditions of applied potential, pH or electrolyte composition^2,5,6,8^. The close onset potentials of OER and catalyst dissolution as well as the characteristic OER over dissolution ratio suggest that the two phenomena are closely linked to each other^2,3,5,8^. This link can be explained by the occurrence of common intermediates for OER and catalyst dissolution^4,8,9^.

Kötz et al. proposed reaction mechanisms considering such common intermediates based on X-ray photoelectron spectroscopy (XPS) investigation of Ruthenium and Iridium oxides. According to the authors, catalyst dissolution on Ruthenium and Iridium oxides occurs during OER with intermediates in oxidation state + 8 ($Ru{O}_{4}$) and + 6 ($Ir{O}_{3}$), respectively^9,10^. However, Iridium accommodating an oxidation state of +3 under oxygen evolution has been reported in different studies supported by XPS^11^ and X-ray absorption spectroscopy (XAS)^12^. Connecting both online inductively coupled plasma mass spectrometry (ICP-MS) and online inductively coupled plasma mass spectrometry (ICP-MS) and online electrochemical mass spectrometry (OLEMS) to a scanning flow cell (SFC) setup, Kasian et al.^4^ proposed a two-pathway dissolution mechanism, assuming a second dissolution path via an OER-intermediate with Iridium in oxidation state +3. The two proposed Iridium oxidation states of the dissolution species follow the thermodynamic stability predictions according to the Pourbaix diagram of Iridium. On the other hand, Rong et al. used ab initio computational methods and discriminated between OER-mechanisms mainly by the occurrence of lattice oxygen participation^13^. The authors suggested that while the so-called adsorbate evolution mechanism determines OER on rutile structured metal oxide surfaces, the so-called lattice oxygen evolution (LOER) mechanism is dominant on amorphous surfaces. In the latter mechanism, the involvement of lattice-oxygen atoms results in weakening of the metal oxide structure, causing catalyst dissolution during OER. The participation of lattice oxygen during OER with Iridium-based catalysts has been confirmed using isotope labelling methods^6,14,15^. Basic thermodynamic reasoning for catalyst dissolution on metal oxides was given by Binninger et al. who showed that, to some extent, dissolution must inevitably occur under OER-conditions due to the thermodynamic instability of the oxygen anion in the metal oxide lattice^8^.

The different proposed mechanisms can be described as a sequence of chemical (C) and electrochemical (EC) reaction steps. The stability behaviour of the reaction system is governed by the common intermediates that either follow the OER-path, as technically desired, or cause catalyst dissolution. In terms of model-based or ab initio theoretical methods, electrochemical Impedance spectroscopy (EIS)^16,17^ and density functional theory (DFT)^13,18^ have been applied to gain insights into the catalytic mechanism of OER. In terms of stability of noble metals, models were developed mainly for Platinum as an oxygen reduction reaction catalyst. Meyers et al. developed a dynamic, microkinetic model for Platinum oxidation and dissolution that includes particle size growth, in which the Pt oxide dissolution via a chemical dissolution step ($PtO + 2{H}^{+}\to {Pt}^{2+} + {H}_{2}O$)^19^ is negligible compared to metallic Pt dissolution via an electrochemical dissolution step ($Pt\to 2{e}^{-}+P{t}^{2+}$). However, the type of dissolution step, either being chemical or electrochemical, is debatable and represents an essential aspect of mechanistic understanding. Based on their model, which predicts the particle radius distribution as well as electrochemical surface area (ECSA) in close agreement to experimental ex situ and in situ studies, Rinaldo et al. suggested that the rate of Pt-O dissolution via a chemical type path may even dominate over the electrochemical type^20^. EIS methods were used for deriving corrosion models of various different metals such as iron or copper^21–23^.

However, so far model-based, microkinetic corrosion studies have focused on potential and pH conditions inside the stability region of water. This is because outside the stability region of water the corrosion current density is hard to deduce, since it is often orders of magnitude smaller than that of the OER. Thus, to derive corrosion models under OER conditions, additional in situ measurement techniques are of essential importance^2^. In recent time, techniques for measuring dissolved species have advanced significantly and provided valuable insights into catalyst stability under OER. However, to the best of our knowledge, so far, no mathematical model has been derived to describe the interplay between OER and catalyst dissolution in a unifying way.

In section “On the role of microkinetic network structure”, based on commonly known concepts of microkinetic theory, the steady-state stability behaviour of a generic electrocatalytic reaction cycle that includes dissolution via a common intermediate is discussed. Although the analysis is exemplified for the OER, the mathematical, microkinetic concepts are developed in a generic form and thus can be directly transferred to different electrocatalytic cycles as well. For instance also for the oxygen reduction reaction (ORR) steady-state dissolution has been reported^24^ and a close link between the two phenomena was proposed^25^. In section “Modelling catalyst dissolution during OER on iridium oxides” the developed concepts are applied to describe the quasi-steady-state OER and catalyst dissolution behaviour on two different Iridium oxide surfaces in acidic media, and the interplay of the two phenomena is discussed.

## On the role of microkinetic network structure

### Tafel-slope analysis

The microkinetic network structure of a reaction mechanism has an important influence on the activity behaviour of an electrocatalytic system. Given a steady-state polarisation curve, a classic method to obtain first insights into the underlying reaction path and its rate-determining step (rds) is the Tafel slope analysis^26^. The idea is to compare the experimentally observed Tafel-slope with those derived from microkinetic models assuming specific reaction steps as the rds. Due to the highly sensitive dependence on potential, in electrochemical kinetics, more likely than in other fields of chemical kinetics, it can be considered that the assumption of one reaction step being much slower than the others is valid, at least over a certain potential range^27^. Two classic assumptions exist for microkinetic modelling of the reaction system, the Quasi-Equilibrium (QE)^27–29^ and the Quasi-Steady-State (QSS)^30^ assumption. The QE-concept is based on the assumption that, due to the slowest reaction step, the overall rate is very slow compared to the rate at which each of the other steps could proceed by itself, and hence the equilibrium of these steps is barely disturbed^27,29,30^. As a consequence, all the reaction steps preceding and following the rds can be assumed in QE (${r}_{i,f}={r}_{i,b}$)^27^. Using the QE-method, the rds is identified or hypothesised a priori before developing a set of model equations.

On the other hand, when only QSS is assumed, the rds is determined by the kinetic reaction constant values of the different steps. Thus, by following the QSS-concept, less assumptions are made during setup of the mathematical model equations. However, more model parameters occur and an explicit expression for the current density cannot be derived. Instead, a system of model equations needs to be solved. When, within the context of a QSS-model, the kinetics of the rds is significantly slower than the kinetics of the other reaction steps (more precisely, forward and backward kinetic constants of that step are at least two orders of magnitude lower than the ones of the other reaction steps), the QSS-model behaves similarly to the QE-model^31^. The consistency of the two concepts can be mathematically shown^30^. A difference in the behaviour of a QSS- to a QE-assumption based model is that the former allows the degree of freedom of changing rds, for instance depending on the potential region. Such change in rds can for instance occur, when the rds is an electrochemical reaction step and the value of its forward kinetic rate constant ${k}_{f}={k}_{f}\left(E\right)$ increases by multiple orders of magnitude, due to the exponential dependence on the potential. It is then possible that the rate constant of the EC-step becomes much higher than the forward kinetic rate constant of another chemical reaction step ${k}_{f,chem}\ne f(E)$, which was fast and fulfilled the QE-assumption at lower potentials. Consequently, the chemical reaction step with the lower rate constant becomes the rds with increasing potential. The simplicity of the QE-concept can be used to obtain all the possible, clearly pronounced Tafel-slopes a given reaction structure can produce by successively assuming different reaction steps as rds^29^. ‘Clearly pronounced’ in this context should be defined as spanning over at least one order of magnitude of current density^32^.

Regarding the different modelling concepts, it was under discussion whether additional clearly pronounced slopes can be observed by assuming only QSS, which do not appear using the QE-assumption^32^. During our simulations we noticed that, independent of QE- or QSS-assumption, it represents an essential difference to either assume the ‘full’ (e.g. 2 and 1) or ‘fractional’ (e.g. 1 and ½) numerical values for the reaction orders with respect to the catalyst surface coverage of species of the forward and backward reaction step, respectively, due to the mathematical non-equivalence of the resulting model equations. Using the ‘full’ reaction orders, identical, clearly pronounced Tafel slopes under both QE- and QSS-assumption are observed. Thus, in terms of reaction activity, classic QE-concept-based Tafel slope analysis remains an important approach that allows discrimination of reaction mechanisms. Naturally, experimental conditions must be designed in a way that non-kinetic effects such as mass transport, pseudo- or double-layer capacitance and changes in effective surface area can be excluded^33^. Table 1 shows the results of the Tafel slope analysis for different OER mechanisms proposed in literature. The slopes in Table 1 are calculated based on the mathematical treatment for microkinetic modelling that have been derived in literature^29,30^. In the following sections, activity is analysed simultaneously with stability using microkinetic theory, which can give valuable insights into the coupling of OER and catalyst dissolution as well as into each of the two phenomena individually.

**Table 1:** Overview of different reaction mechanisms and derived Tafel slopes for OER and Langmuir kinetics.

	OER-path	Sequence (1, 2, 3, …)	(rds) Slopes [mV/dec]			Source(s)
1	DFT-predicted peroxide path, ‘acid base’	EC, EC, EC, EC	(4) 17, 24, 40, 120 (3) 24, 40, 120	(2) 40, 120 (1) 120		^18,34^
2	Shinagawa	EC, EC, EC, C, EC	(5) 17, 14, 40, 120 (4) 20, 30, 60, ∞	(3) 24, 40, 120 (2) 40, 120	(1) 120	^28^
3	Kobussen	EC, EC, C, EC, EC	(5) 17, 24, 40, 120 (4) 24, 40, 120	(3) 30, 60, ∞ (2) 40, 120	(1) 120	^35^
4	“Direct coupling”	EC, C(AA), C, C, EC, EC	(6) 17, 40, 120 (5) 24, 120	(4) 30, ∞ (3) 30, ∞	(2) 30, ∞ (1) 120	^34^
5	Electrochem. oxide path, O ‘Grady’ s path	EC, EC, C(AA)	(3) 15, 30, ∞ (2) 40, 120	(1) 120		^36^
6	Krasil ‘shchikov’ s path	EC, C, EC, C(AA)	(4) 15, 30, ∞ (3) 40, 120	(2) 60, ∞ (1) 120		^37^
7	Oxide path	EC, C(AA), C(AA)	(3) 7.5, 15, ∞ (2) 30, ∞	(1) 120		^36^
8	Electrochemical metal peroxide path	EC, C(AA), C, C(AA)	(4) 7.5, 15, ∞ (3) 30, ∞	(2) 30, ∞ (1) 120		^36^
9	Ir(III)-path	C, EC, C(AA), EC	(4) 20, 120 (3) 30, ∞	(2) 120 (1) ∞		^4^
10	$Ir{O}_{3}$-path	C, EC, EC, C(AA)	(4) 15, 30, ∞ (3) 40, 120	(2) 120 (1) ∞		^9^
11	Physisorbed peroxide path	EC, EC, C, C(AB)	(4) 15, 30, ∞ (3) 30, 60, ∞	(2) 40, 120 (1) 120		^33^
12	Hydrogen peroxide path Metal peroxide path	EC, C(AB), C(AB), C(AB)	(4) 15, ∞ (3) 20, ∞	(2) 30, ∞ (1) 120		^36^

Calculation of the slopes is based on theory by Gileadi^27^ and Bockris et al^29^ (given for room temperature). The numbers in parenthesis denote the position of the assumed rds. The values to the right of the parenthesis represent the possible, clearly pronounced Tafel slopes associated with the assumed rds. Higher Slope values further to the right of the parenthesis correspond to the saturation of coverage by later species in the reaction cycle. AA and AB denote dimerisation steps with similar and different reactant species, respectively.

### OER over dissolution selectivity

By considering catalyst dissolution as an undesired side-reaction, the definition of an OER over dissolution selectivity, in the following simply referred to simply as ‘selectivity’, can be used to describe electrocatalytic stability. As the mathematical definition the so-called stability number (${S}_{num}$) shall be chosen herein, which is defined as the ratio of oxygen molecules produced per catalyst atom dissolved^6^.

$${S}_{num}=\frac{{r}_{OER}}{{r}_{diss}}$$

**Figure 1:**
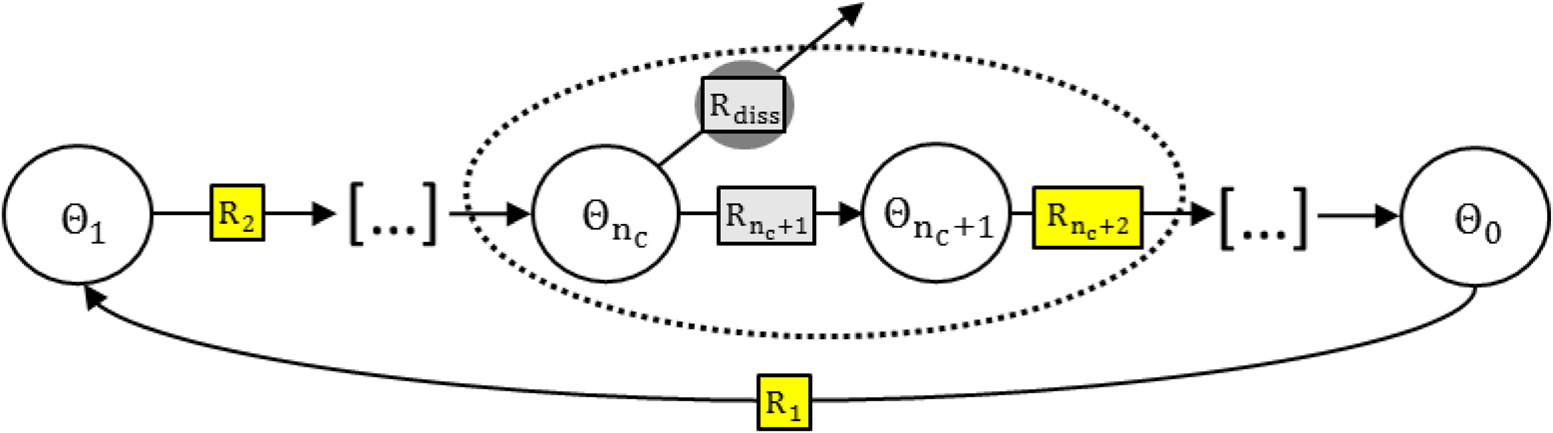
Generic description of an electrocatalytic reaction cycle with dissolution of an intermediate species. Reaction steps including electron transfer are coloured in yellow, while the grey reaction steps are considered either of C- or EC-type. ${\Theta }_{0}$ denotes the state occurring after rds. For the analysis of binuclear mechanisms, one of the reaction steps ${R}_{i}$ is considered as a dimerisation step. ${n}_{c}$ denotes the index of the common intermediate.

In the following sections, the influence of the microkinetic network structure on selectivity is discussed. For the mathematical expressions, the QE-assumption is used as a basis. Thus, the derived expressions can be used when the QE-assumptions are fulfilled for the reaction system. However, it may be the case that the QE-assumption is not fulfilled for all reaction steps. Thus, conclusions that are valid not only under assumption of QE will be particularly pointed out in the following sections. Figure 1 shows the representation of a generic catalytic reaction cycle that includes catalyst dissolution via a common intermediate. In Figure 1 except from the rds and the dissolution step, only electrochemical type reaction steps are considered since, following the QE-concept, for a mononuclear, chemical reaction step in QE, the ratio of reactant and product species is determined by a potential-independent equilibrium constant $K\ne K(E)$. Thus, from a mathematical point of view these chemical steps in QE do not contribute additional features to the possible behaviour of the microkinetic structure. On the other hand, chemical, binuclear reaction steps (in QE or non-QE) change the behaviour of the microkinetic structure and thus will be discussed further in section “Bi-nuclear mechanism with rds as dimerisation step”.

In the following sections, it is analysed how the microkinetic network structure determines the behaviour of the selectivity depending on the potential. The possibility of redeposition shall be included in the analysis by introducing a variable $\vartheta$ as the ratio of (forward) dissolution and (backward) redeposition reaction rate. Defined in this way, $\vartheta$ is potential dependent for an electrochemical dissolution step. However, in the two extremal potential regions $\vartheta$ does not change with potential $(d\vartheta /dE\approx 0)$, i.e. the redeposition step is negligible ($\vartheta \approx 0$) or the dissolution step is in QE ($\vartheta \approx 1$). At a given potential, the experimental conditions determine the relevant case e.g. with variation of the flow rate or the experimental time. The ideal case $\vartheta =1$ corresponds to very long experimental times for batch experiments or very low flow rates in SFC experiments. In this case the rate of dissolution is equal to the rate of redeposition^38–40^. On the other hand, the ideal case $\vartheta =0$ corresponds to short experimental times or high flow rates in batch or SFC experiments, respectively, where the redeposition rate is negligible compared to dissolution rate. In order to gain mechanistic insights into pathways, it is beneficial to decouple effects by choosing experimental conditions at which $\vartheta$ is constant. However, experimental constraints such as detection limits of the analytical methods must naturally be considered as well.

#### Mononuclear mechanism

Depending on $\vartheta$, an expression of ${S}_{num}$ for mononuclear mechanisms can be derived (Supplementary Information):

1 $${S}_{num}=exp\left(\left[{n}_{EC}+\left({z}^{rds}-{z}^{diss}\left(1+\vartheta \right)\right)\beta \right]f E\right) \frac{{k}_{rds}}{{k}_{diss}} \prod_{j={n}_{c}}^{{n}_{rds}-1}{K}_{j}$$

${\text{n}}_{\text{EC}}$ denotes the number of electrochemical reaction steps between the common intermediate and the direct reactant of the rds. z = 1 is considered if the reaction step is of electrochemical type and z = 0 for a chemical type. The derivative of the selectivity ${S}_{num}$ with respect to potential can then be calculated as:

2 $$\frac{{{\text{d}}{{\rm{S}}_{{\text{num}}}}}}{{{\text{dE}}}} = \exp \left( {{{\rm{Z}}_1}{\text{fE}}} \right) \cdot {{\rm{Z}}_1} \cdot {\rm{f}}\frac{{{{\rm{k}}_{{\text{rds}}}}}}{{{{{{\bar{\text{ k}}}}}_{{\text{diss}}}}}}\prod\limits_{{\text{j}} = {{\text{n}}_{\text{c}}}}^{{{\text{n}}_{{\text{rds}}}} - 1} {{{\text{K}}_{\text{j}}}}$$

$${Z}_{1}$$ represents the term in brackets in Eq. (1). Since the exponential function in Eq. (2) is always positive, the sign of $$d{S}_{num}/dE$$ is dictated by $${Z}_{1}=f\left({\text{n}}_{\text{EC}}\text{, }{\text{z}}^{{\text{r}}\text{ds }}\text{, }{\text{z}}^{\text{diss}}\right)$$. Thus, Eq. (2) indicates that, the behaviour of the selectivity (constant, increase or decrease), depends on the microkinetic network structure under given experimental conditions ($$\vartheta =0\ {\text{or}}\ \vartheta =1)$$. Table 2 shows the analysis performed using Eq. (2) for twelve different cases considering either direct competition of dissolution and rds ($${n}_{rds}={n}_{c+1})$$ or one more EC-step in QE between the common intermediate and the rds ($${n}_{rds}={n}_{c+2})$$. The considered part of the network structure in Fig. 1, which is described by Table 2, is circled with a dotted line.

**Table 2:** Dependence of selectivity on the electrode potential of different reaction step arrangements based on Eqs. (2) and (4).

No	${n}_{RDS}$	${r}_{{n}_{c}+1}$	${r}_{{n}_{c}}$	${r}_{diss}$	Sign $(d{S}_{num}/dE)$	
					$\vartheta =0$	$\vartheta =1$
1	${n}_{c}$	n/r	C	C	[0]	[0]
2	${n}_{c}$	n/r	C	EC	−	−
3	${n}_{c}$	n/r	EC	EC	0	−
4	${n}_{c}$	n/r	EC	C	+	+
5	${n}_{c+1}$	C	C	C	[0]	[0]
6	${n}_{c+1}$	C	C	EC	−	−
7	${n}_{c+1}$	C	EC	EC	+	0
8	${n}_{c+1}$	C	EC	C	+	+
9	${n}_{c+1}$	EC	C	C	+	+
10	${n}_{c+1}$	EC	C	EC	0	−
11	${n}_{c+1}$	EC	EC	EC	+	+
12	${n}_{c+1}$	EC	EC	C	+	+

The behaviour of d ${S}_{num}$/dE is shown for a mononuclear rds. A binuclear (quadratic reaction order) rds in the special case of saturation at high potentials $({\theta }_{{n}_{rds}}\to 1$) behaves similarly. Two cases are considered for the forward and backward direction of the dissolution step: ${r}_{diss,f}\gg {r}_{diss,b}$ ($\vartheta =0$) and Quasi-Equilibrium ${r}_{diss,f}\approx {r}_{diss,b}$ ($\vartheta =1$). For a binuclear rds, the results marked with brackets, [], occur under limiting current conditions due to a slow chemical reaction step. The reaction steps marked with n/r are not relevant for the selectivity description.

#### Bi-nuclear mechanism with rds as dimerisation step

In contrast to the mononuclear case where only a single site is involved, in binuclear reaction mechanisms there is (at least) one reaction step that involves two adjacent reaction sites. In many OER-mechanisms proposed in literature, a reactant needs to be formed twice such that the reaction can further proceed. For this case the reaction step is expected to show an over proportional dependence on its reactant site coverage and a quadratic reaction order is typically assumed $(\nu =2)$. Thus, the selectivity can be described for a bi-nuclear rds by (derivation see Supplementary Information):

3 $${S}_{num}=\frac{{\left(\prod_{j=1}^{{n}_{rds}-1}{K}_{j}\right)}^{2}{k}_{rds} }{{\stackrel{-}{k}}_{diss}\left(\prod_{j=1}^{{n}_{c}}{K}_{j}\right)\left(\sum_{m=0}^{{n}_{rds}}exp\left([m-{n}_{rds}-{n}_{EC}+\beta \left({z}^{diss}\left(1+\vartheta \right)-{z}^{rds}\right)\right]f E)\prod_{j=1}^{m+1}{K}_{j}\right)}$$

Using the chain rule, the derivative with respect to potential is obtained:

4 $$\frac{d\left({S}_{num}\right)}{dE}=-\frac{1}{{D}^{2}}{\left(\prod_{j=1}^{{n}_{rds}-1}{K}_{j}\right)}^{2}{k}_{rds} {\stackrel{-}{k}}_{diss}\prod_{j=1}^{{n}_{c}}{K}_{j}\cdot \sum_{m=0}^{{n}_{rds}}\left(exp\left({Z}_{2} f E\right)\cdot {Z}_{2}\cdot f\prod_{j=1}^{m+1}{K}_{j}\right)$$

D denotes the denominator of Eq. (3) and ${Z}_{2}$ the term in brackets in Eq. (3). The resulting expression in Eq. (4) shows that, again, the sign of $d{S}_{num}/dE$ is determined by ${{Z}_{2} =f(m, n}_{rds},{n}_{EC}, {z}^{diss},{z}^{rds})$. Thus, for given experimental conditions ($\vartheta =0\ {\text{or}}\ \vartheta =1)$ the sign of $dS_{num/dE}$ is determined by the microkinetic network structure. The results for the considered binuclear rds and the 12 different reaction structures as circled in Fig. 1 are shown in Table 2 for very high potential values $({\theta }_{{n}_{rds}}\to 1$).

#### Bi-nuclear mechanism when dimerisation step is not the rds

When the dimerisation reaction step occurs before the common intermediate, it has no effect on the selectivity, as Eq. (3) does not include reaction steps before the common intermediate. The same holds for a dimerisation step after the rds, which only changes selectivity by the factor 0.5, due to the reaction stoichiometry. For the case that the dimerisation reaction step is located at a position after the common intermediate and before rds $({n}_{c}<{n}_{dim}<{n}_{rds})$, it does not seem to derive a compact mathematical expression that covers all different possible cases. However, it can be noted that, due to the quadratic dependence of the forward reaction rate of the dimerisation reaction step, a higher sensitivity of the OER rate and shift of coverage on potential can be expected compared to the same reaction sequence but with the reaction order $\nu =1$. When the sensitivity of the OER rate on potential is higher than the sensitivity of the dissolution step (Tafel slope is lower than the slope of dissolution), the selectivity increases with potential.

#### Discussion on selectivity

Equations (2) and (4) show that the more EC-steps are located between $${\Theta }_{{n}_{c}}$$ and $${\Theta }_{{n}_{rds}}$$ (the higher $${n}_{EC})$$ the more positive is the dependence of selectivity on potential. On the other hand, an EC-type dissolution step contributes to a more negative dependence. An interesting prediction of Eqs. (2) and (4) is that a chemical type dissolution reaction step ($${z}_{diss}=0$$) cannot lead to a decreasing selectivity with increasing potential, independent if the OER-mechanism is mono- or binuclear. Adding the condition that the competing OER reaction step is electrochemical, the selectivity increases (Table 2). For mononuclear mechanisms these two conclusions are independent of the QE-assumption. A simple derivation without using the QE-assumption is shown in the Supplementary Information. For mononuclear mechanisms and the simple cases of direct competition of dissolution step with the rds (Table 2, rows 1–4), Eq. (2) predicts that the selectivity is constant when the competing reaction steps are of same type ($$E{C}_{diss}/E{C}_{rds}$$ or $${C}_{diss}/{C}_{rds}$$). When only one of the competing reaction steps is electrochemical, with increase in potential the reaction is driven in favour of this reaction step with potential increase, thus a change in selectivity can be expected. The conclusion for the cases of direct competition of rds with the dissolution step (cases 1–4) are independent of the QE-assumption. A derivation of the selectivity expression for these cases without using the QE-assumption is shown in the Supplementary Information.

The sign of $d{S}_{num}/dE$, as shown in Table 2, is the same with both mono- and bi-nuclear rds at very high potentials $({{n}_{rds}}\to 1$), since the effect of the quadratic reaction order diminishes with a constant coverage ${\Theta }_{{n}_{rds}}$. For chemical type rds, limiting current conditions occur. For a binuclear rds, in all considered cases shown in Table 2 it is possible, depending on the parameterisation of the model kinetics, that the network structure shows an initial increase of the selectivity with potential. This is because, depending on the kinetic parameters, the effect of the quadratic reaction order towards OER may exceed the potential dependence of the dissolution reaction. In the case that the rds is binuclear, Eq. (4) indicates that the selectivity only decreases with potential, when the dissolution step is electrochemical and there is no EC-step between common intermediate and rds, the rds itself included (${z}_{diss}=1, {n}_{EC}=0$ and ${z}_{rds}=0$). This case is represented in Table 2 by cases 2 and 6.

In the presented analysis, Langmuir conditions are considered. Thus, the free energy of adsorption is assumed as constant^27^. Temkin adsorption kinetics can account for the change of the free energy of adsorption. When the effects of surface inhomogeneity or lateral repulsive interactions dominate (Temkin parameter $${\text{r}}_{\text{Tem}}\text{ } > \text{ 0}$$) the shift in coverage is less sensitive to potential and the forward reaction of OER is increased less with potential compared to the Langmuir case^27^. Therefore, it can be expected that, if an OER reaction step between common intermediate and rds is controlled by Temkin adsorption kinetics, d $${S}_{num}$$/dE decreases with increasing potential compared to the Langmuir case. The opposite can be expected for dominating later, attractive interactions ($${r}_{Tem}<0)$$.

### Dissolution rate

Analogous to Tafel-slope analysis for OER activity, calculation of ‘slope of dissolution’, which describes the potential dependence of the measured dissolution^41^, as well as evaluation of reaction orders e.g. regarding proton activity, can provide valuable mechanistic insights. Furthermore, considering that the QE-assumption is fulfilled, the coverage monotonously shifts towards the species in later position of the reaction cycle with increasing potential^26^. When dissolution occurs via a chemical step, the dissolution rate can be expected proportional to the coverage of the common intermediate. Thus, under the QE-assumption, when the common intermediate is the direct precursor or the direct product species of the rds, the dissolution rate is expected to increase or decrease with increase in potential, respectively. This is the case, for instance, when the QE-assumption is made for the dissolution mechanism via Ir(III)^4^ and the oxygen recombination step is considered as rds^42^. This mechanism will be further discussed in section “Reactively sputtered Iridium oxide surface”.

## Modelling catalyst dissolution during OER on iridium oxides

In the following sections, microkinetic modelling is applied to analyse catalyst dissolution during oxygen evolution on Iridium oxides in acidic media. Two Iridium oxide surfaces are studied: The thermally treated (TO) and reactively sputtered (RS) Iridium oxide surfaces. The TO type of surface is obtained by thermal preparation at elevated temperatures which leads to a rutile structured, Iridium oxide with a stoichiometry of $Ir{O}_{2}$^1,4^. In contrast to hydrous Iridium oxide ($Ir{O}_{x}$), the RS-surface incorporates a similar surface stoichiometric composition as the TO–$Ir{O}_{2}$^4^- However, compared to TO-$Ir{O}_{2}$, the RS-surface shows a lower degree of crystallinity, more lattice imperfections and a less well-defined lattice termination^43^, since no thermal preparation step is included. Therefore, surface structure and morphology are important differences between the two electrocatalytic surfaces considered in the following sections. After analysing the characteristic features from published experimental data and accounting for insights provided by ab initio DFT-studies, models are derived to describe the quasi-steady-state OER-dissolution behaviour without a priori quasi-equilibrium assumption. The models are then compared with experimental observations and discussed.

### Thermal iridium oxide

#### Microkinetic analysis of OER and dissolution on thermal iridium oxide

**Figure 2:**
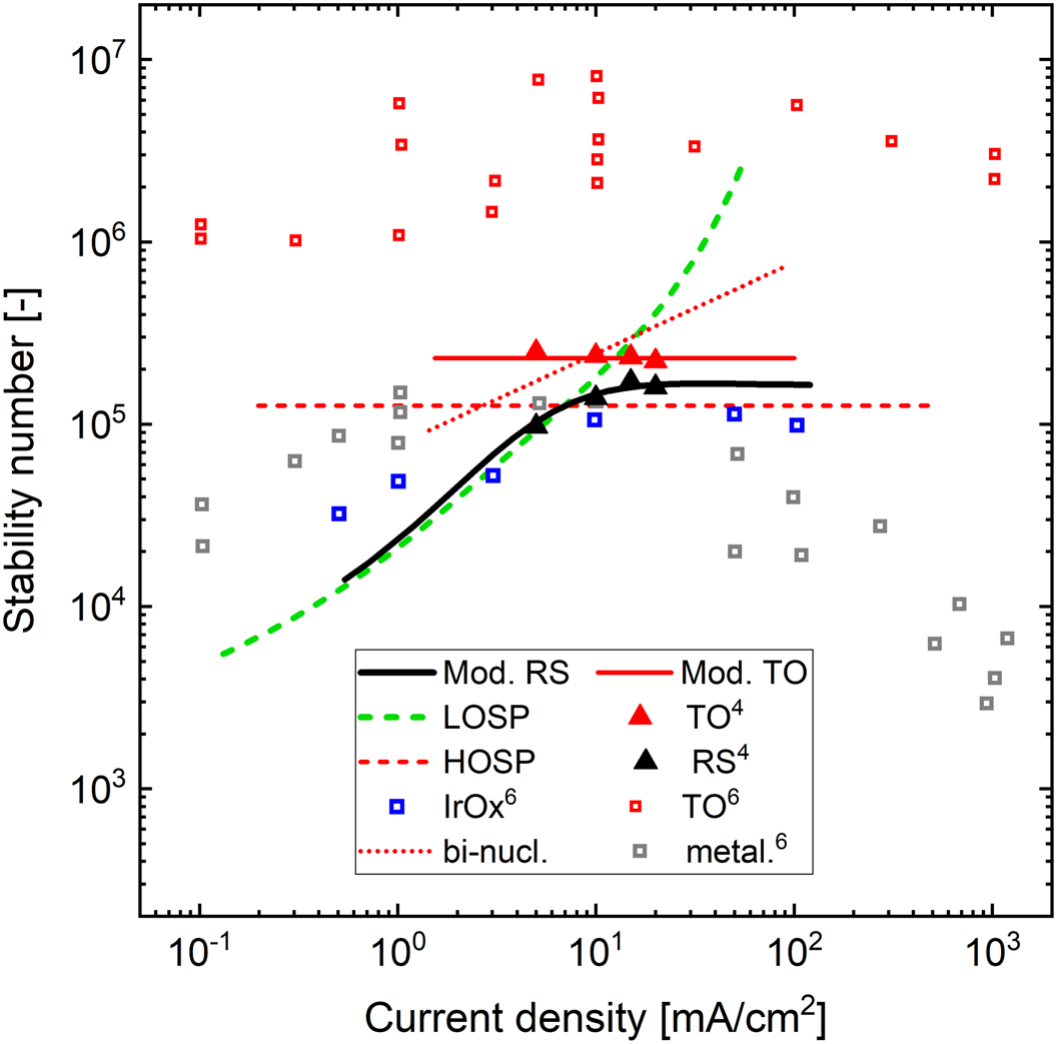
Stability number of different Iridium based OER catalyst films. The open^6^ and filled symbols^4^ show experimental data, whereas model predictions are represented by lines. The thermal oxide (TO) model (structure in Fig. 3c) is shown by the red, solid line. The bi-nuclear mechanism via coupling of two adjacent $Ir{O}_{3}$ species (structure in Fig. 3b) is presented by the red, dotted line. The RS-model results are shown along with those of the individual paths, as derived in section “Reactively sputtered Iridium oxide surface”. Additionally, experimentally observed data of metallic Iridium and hydrous Iridium Oxide (${IrO}_{x}$) are shown^6^.

In different experimental studies with rutile $Ir{O}_{2}$ surfaces, the measured dissolution data indicates a characteristic proportionality between dissolution rate and current density (Fig. 2)^3,4,6^. Therefore, the potential dependence (slope) is similar and the selectivity is constant. Under well-defined conditions, as discussed previously, the experimentally observed, clear Tafel slope values are around 60 mV/dec at room temperature in acidic media^1,3,32,44–48^. These characteristics must be reflected in a suitable mechanistic model.

As mentioned before, LOER has been suggested to explain the higher OER activity and catalyst dissolution on amorphous metal oxide structures. According to this mechanism, the removal of a lattice oxygen atom during OER creates an oxygen vacancy. This defect of the lattice structure is either ‘repaired’ by a re-oxidation step or it can cause dissolution of Iridium atoms. It is under discussion whether some smaller fraction of LOER is also the main mechanism for dissolution on rutile Iridium oxides, suggesting its universal character, or other dissolution pathways and intermediates are involved^6^.

**Figure 3:**
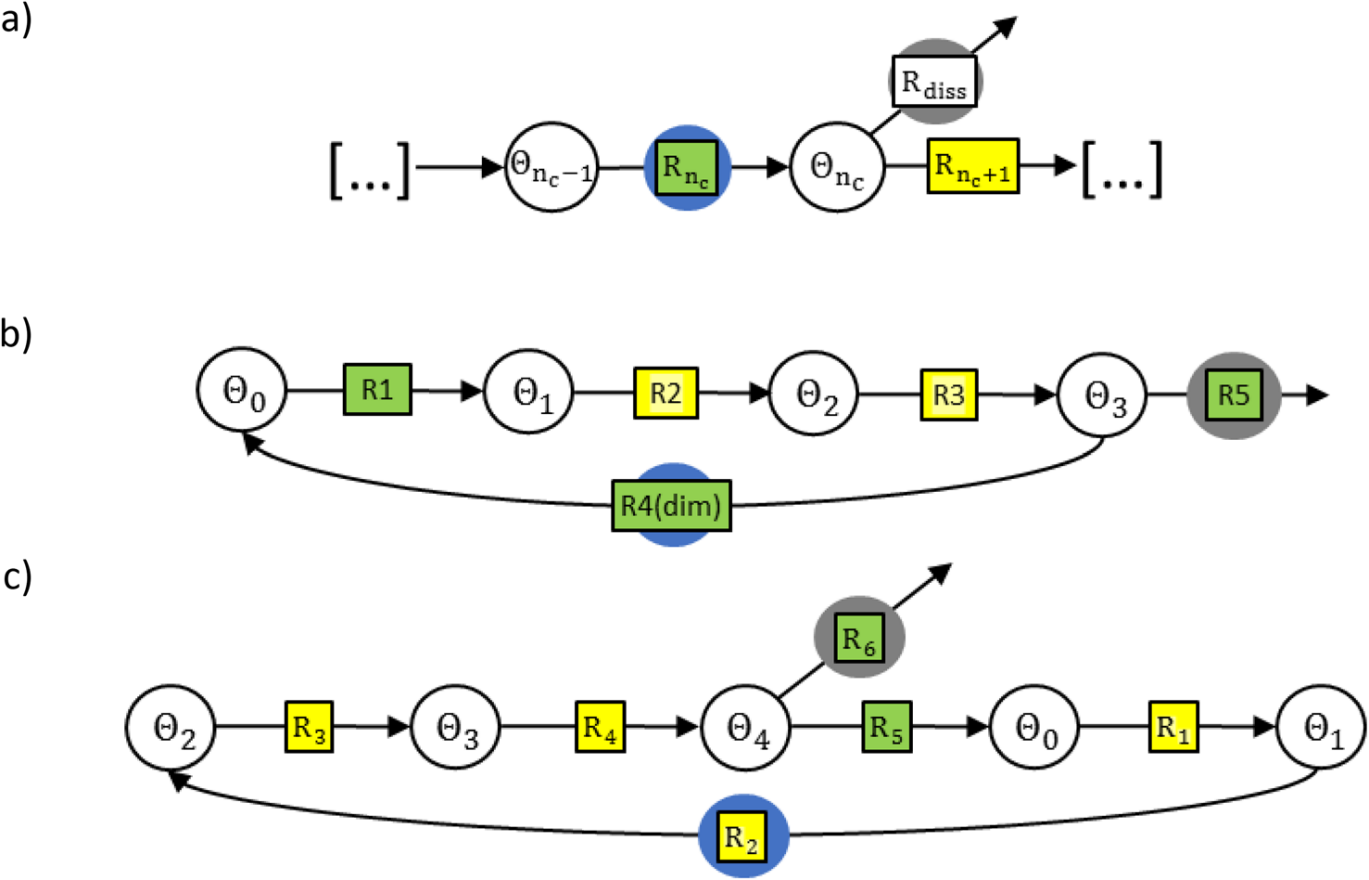
Microkinetic network structure of different OER-dissolution mechanisms consisting of chemical (green) and electrochemical (yellow) reaction steps. Blue and grey circle mark oxygen evolving and dissolution reaction step, respectively. (**a**) Sequence of the lattice oxygen evolution mechanism^6,13^. For the dissolution step (shown with a white box) the type (C or EC) is discussed. (**b**) $Ir{O}_{3}$-path via coupling two oxygen from adjacent sites^4,9^ (**c**) Model reaction network structure based on the adsorbate evolution mechanism via mononuclear ‘acid–base’ path^6,34^ and a chemical rds^35^. Possible speciation of the reaction intermediates corresponding to the coverages ${\Theta }_{i}$ are given in Supplementary Tables S1 and S2 based on literature suggestions.

The characteristic dissolution sequence of the LOER mechanism is provided in Fig. 3a. When a lattice oxygen vacancy is formed during LOER, its re-oxidation is in competition with a dissolution reaction step of either chemical ($${C}_{diss}/EC)$$ or electrochemical type $$(E{C}_{diss}/EC)$$^8^. As discussed in section “OER over dissolution selectivity”, due to the EC-type re-oxidation step, a chemical dissolution step results in an increase in selectivity with potential in contrast to experimental observations (Fig. 2). During the loss of a lattice oxygen atom, the neighbouring Iridium atom(s) can be assumed in an oxidation state lower than + 4. It seems unlikely that (i) such an Iridium atom dissolves via electrochemical step, e.g. following the Pourbaix diagram of Iridium via multiple electron transfer steps to + 6 ($$Ir{O}_{4}^{2-}$$)^49^, and (ii) the combination of OER steps from common intermediate to rds is ‘coincidently’ characterised by an identical electrochemical dependence (slope) as the dissolution reaction. Thus, in the following model derivation, it is assumed that the dissolution on rutile Iridium oxides is not explained by a LOER-mechanism but occurs via a fundamentally different pathway. Studies have suggested an OER-dissolution path via formation of volatile $$Ir{O}_{3}$$^4,9^, which shall be further analysed in the next section.

Figure 3b shows the network structure of the mechanism via $Ir{O}_{3}$, which was proposed for rutile Iridium oxide^4,9^, due to the mass spectrometric detection of dissolving species with mass to charge ratio of 240, associated with $Ir{O}_{3}$. In terms of microkinetic network structure, a characteristic property of this mechanism (Fig. 3b) is that a chemical, mononuclear dissolution step is in direct competition with the direct coupling of two adsorbed oxygen atoms to release an oxygen molecule (${C}_{diss}/C(AA))$. However, as discussed in section “OER over dissolution selectivity”, this network structure results in either increasing selectivity or limiting current conditions, both not observed experimentally. In addition, the microkinetic model of the reaction network in Fig. 3b does not reproduce a Tafel slope of ca. 60 mV/dec, due to the binuclear oxygen recombination step. For visualisation, Fig. 2 shows the selectivity behaviour predicted by microkinetic modelling of the bi-nuclear mechanism corresponding to Fig. 3b in comparison to the experimental data.

**Figure 4:**
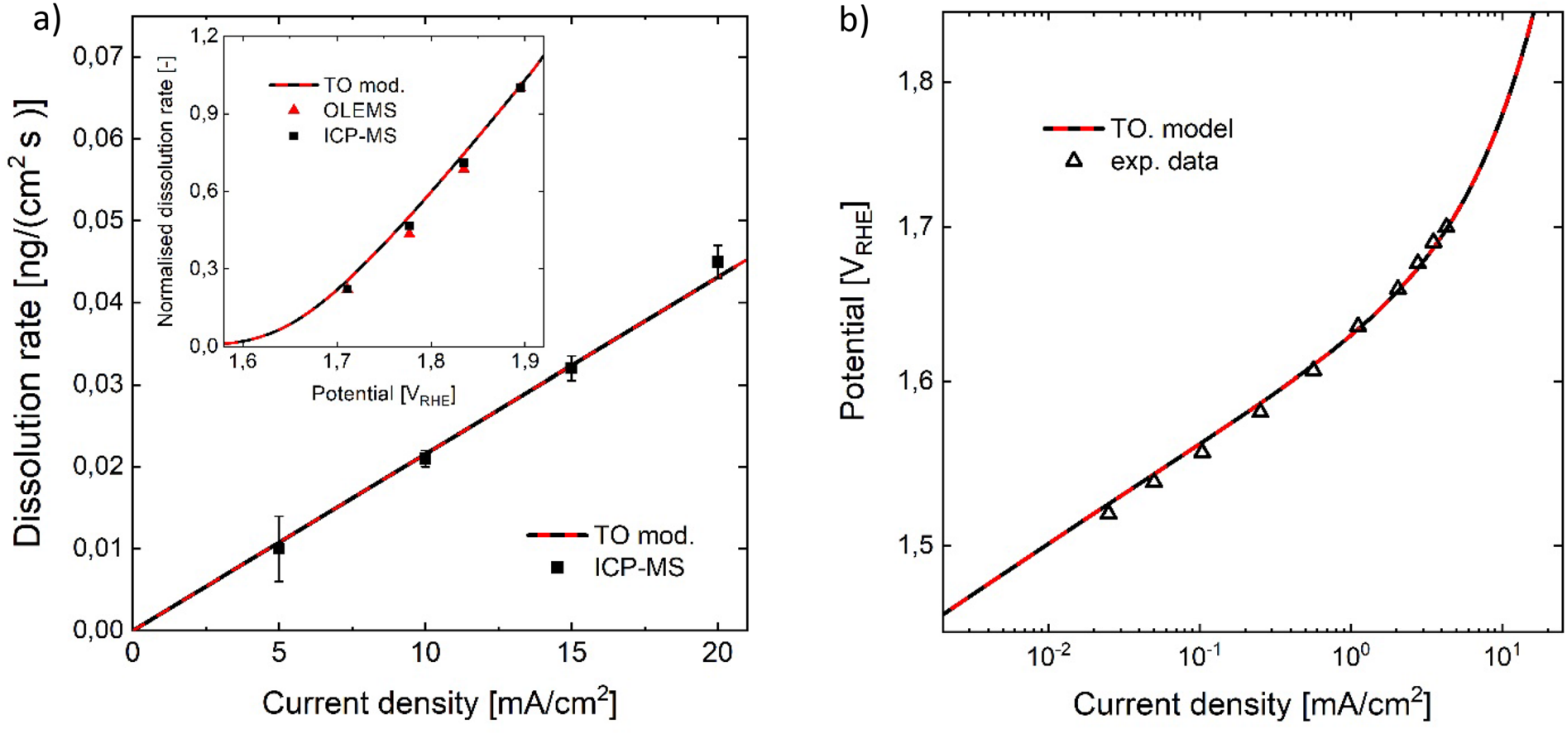
Comparison of model of thermal oxide $Ir{O}_{2}$ with experimental data, measured with a flow cell setup^3,4^. (**a**) Comparison with dissolution rates measured with inductively coupled plasma mass spectrometry (ICP-MS) and online electrochemical mass spectrometry (OLEMS) in 0.1 M $HCl{O}_{4}$, showing a linear correlation of OER and dissolution rate. Inset: Model compared to ICP-MS and OLEMS dissolution rate signals, both normalised by dividing by the highest dissolution value. (**b**) Comparison with experimental electrochemical data^3^, which shows a characteristic slope of ca. 60 mV/dec at room temperature in acidic media ^1,3,32,44–48^.

In line with XPS analysis^50^, DFT-studies have suggested that a sequential water dissociation path, in which an ${O}-{O}$ bond is formed on a single surface site is more favourable for both $Ir{O}_{2}$^47,51^ and $Ru{O}_{2}$^52^. Thus, in the following sections an adsorbate evolution type mechanism following the mononuclear, ‘acid–base’ path shall be assumed for modelling the TO-surface^6^. The considered microkinetic structure, which is shown in Fig. 3c, is similar to that proposed by Kobussen et al.^35^. Following previous studies it is assumed that dissolution occurs via a chemical reaction step after the adsorption of an oxygen atom on the $Ir{O}_{2}$ site^4,9^. An overview of the reaction equations and intermediates that can be assigned to the considered network structure is given in the Supplementary Tables S1 and S2. To account for increasing variation of adsorption conditions with coverage due to surface morphology and heterogeneity, Temkin adsorption kinetics are considered for the ${O}$ formation step. Accounting for flow conditions in an SFC-setup, a negligible backward reaction (redeposition) is assumed. For parameterisation of the kinetic model three types of experimental data were fitted simultaneously. The ICP-MS data is considered as the total dissolution rate, while the OLEMS signal with a mass-to-charge ratio of 240 (m/z) is assigned to volatile $Ir{O}_{3}$^4^. Since no quantitative dissolution rates can be derived from the measurements of volatile $Ir{O}_{3}$, the normalised OLEMS signal with respect to the highest dissolution rate was included into the model fit. Considering the experimental data, the assumption of a single dissolution path is possible for modelling the rutile $Ir{O}_{2}$ surface, only due to the fact that the normalised data of liquid-dissolved and volatile species highly overlap (inset Fig. 4a).

#### Model discussion

The simulation data (Fig. 4a, b) show close agreement with the experimental measurements. The experimentally observed Tafel slope of around 60 mV/dec is reproduced, due to the assumption of a proton-electron decoupled reaction step^53^ ($S$–$O$ + ${H}_{2}O$  → $S$–$OO{H}^{-}+{H}^{+}$), in which the slow chemical adsorption is the rds and precedes the fast electron removal. In combination with the formation of $S-O$ in quasi-equilibrium, the mononuclear $EC-{C}_{rds}$ OER sequence reproduces the observed slope. The constant selectivity shown by experimental data is explained by the competition of rds with dissolution ($Ir{O}_{2}$–$O$  → $Ir{O}_{3}$), both of chemical type $({C}_{diss}/{C}_{rds})$. The rate-determining chemical adsorption of the second hydroxyl group^35^ appears in line with XAS-studies, which have indicated that adsorbed, reactive and electrophilic ${O}^{I-}$ species are directly correlated with OER activity^42^ and thus can be interpreted as the reactant of the rds. Furthermore, DFT-studies have pointed out that the formation of the ${-OOH}$ intermediate, following the adsorption of ${O}$, is the energetically least favourable reaction step^51,54,55^.

Analysis of the proton reaction order can further support discrimination of mechanistic pathways. Since the backward reaction of the rds is negligible, the step is unaffected by the proton activity. The considered dissolution step ($Ir{O}_{2}-O$ → $Ir{O}_{3}$) is also unaffected by proton activity. On the other hand, the quasi-equilibrated formation of $O$ incorporates a mechanistic proton reaction order (at constant potential versus standard hydrogen electrode) of −1 since a proton is involved as a product species. Therefore, the model of TO–$Ir{O}_{2}$ predicts a reaction order of −1 for both OER and dissolution below ca. 1.5 ${V}_{RHE}$. At higher potentials, the reaction order tends to zero as the theoretical limiting current conditions are approached (Supplementary Fig. 1). Studies in acidic media have reported proton reaction orders of −1 for thermally prepared $Ir{O}_{2}$^48^ and − 0.75 for thermally prepared 90% $Ir{O}_{2}$ with 10% $Sn{O}_{2}$^56^. A comparative study on thermal $Ir{O}_{2}$ has shown dissolution rates of 0.07 and 0.15 ng/cm^2^ in 0.1 M $HCl{O}_{4}$ and 0.05 M $NaOH$, respectively, during sweeping in OER conditions^3^. Considering the many orders of magnitude difference in proton activity, the observed, relatively small difference suggests a proton reaction order of the dissolution rate close to zero for constant potential E vs. RHE, thus a mechanistic reaction order close to −1 as predicted by the model. Lower OER activities observed in alkaline media were proposed to be related to the observation of a non-Nernstian decrease of $O$ and $OH$ adsorption potentials vs ${E}_{RHE}$ towards higher pH, which in turn causes an increasingly unfavourable $OOH$ formation^45^. It may be hypothesised that, while the OER mechanistic path changes towards utilisation of $O{H}^{-}$ for alkaline conditions^45^, the dissolution path via adsorbed $O$ is similar for acid and base. Overall, due to its microkinetic structure, the derived model shows good agreement with different experimental observations of activity and stability of rutile $Ir{O}_{2}$ in acidic media.

It is noted that from a mathematical point of view, the model fit works equally well with the assumption of the dissolution step in QE with the electrolyte ($\vartheta =1$), when applying the concept of an apparent kinetic constant ${k}_{diss}^{app}=f\left(\dot{V}, {A}_{electrode}\right)$ for qausi-equilibrated electrolyte solution as described in the Supplementary Information. It is suggested that the variation of the flow rate or experimental time, in flow-cell or batch experiments, respectively, or using different electrolyte anions, which can affect equilibrium conditions due to complexing^49^, can help for discriminating between the cases $\vartheta =1$ and $\vartheta =0$. It is also noted that, despite fitting the model to three different types of (electro-)chemical data, identifiability analysis reveals that no uniqueness is obtained for the whole parameter set, in line with with mathematical studies on model parameter identifiability of electrochemical reaction systems using steady-state methods^57^. However, the aim of this work is not the extraction of kinetic parameter values, but rather to gain mechanistic insights via simultaneous analysis of different types of experimental data and characteristic microkinetic sequences.

### Reactively sputtered iridium oxide surface

#### Microkinetic analysis of OER and dissolution on reactively sputtered iridium oxide

**Figure 5:**
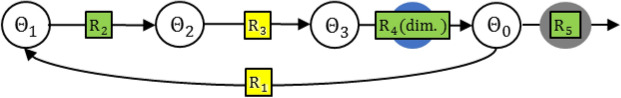
Microkinetic network structure of the reaction path via Ir(III)^12^ as common intermediate for OER and catalyst dissolution^4^. Possible speciation of the reaction intermediates corresponding to the coverages ${\Theta }_{i}$ are given in Supplementary Table S2.

**Figure 6:**
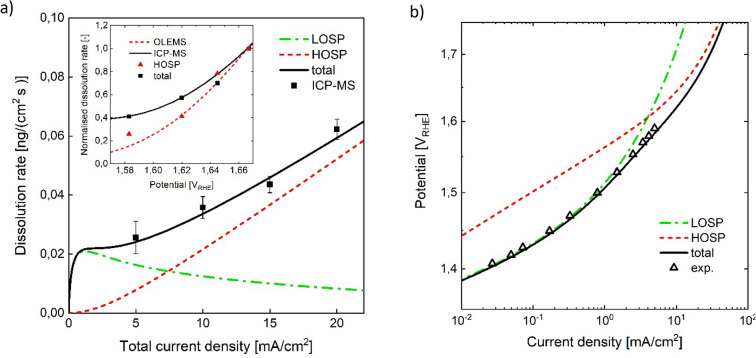
Comparison of model with experimental data, measured in a flow cell setup coupled with ICP-MS and OLEMS in 0.1 M $HCl{O}_{4}$^4,15^. (**a**) Comparison of model to measured liquid dissolved dissolution rate, plotted over overall current density. Inset: Model and measured signals of liquid dissolved and volatile Iridium dissolution normalised by dividing with the value at the highest current density, are plotted over potential. (**b**) Model and electrochemical data of reactively sputtered iridium oxide^15^. HOSP and LOSP denote high and low oxidation state path considered in the RS-model, respectively.

In terms of both activity and stability in acidic media, a qualitatively different behaviour is observed with a reactively sputtered (RS) Iridium oxide^4,58,59^ compared to thermal Iridium oxide^4,6,15^. Analysis of the dissolution data of the RS-surface (inset Fig. 6a) shows, in contrast to that of the TO-surface, a significant difference of onset potentials for liquid-dissolved and volatile dissolution rates. To capture this discrepancy, a mechanistic model must incorporate at least two dissolution pathways. In previous studies it was proposed that the more active electrocatalytic surfaces undergo OER via transition over a low oxidation state intermediate^4,12^, which might include the formation of a lattice oxygen vacancy via LOER^6,13^. The ratio of OER undergoing the one or the other type of mechanism has been proposed to govern the behaviour of electrocatalytic activity as well as stability^4,6,13^. Based on this hypothesis, in the following a quasi-steady state model for a surface of reactively sputtered $$Ir{O}_{2}$$ is derived, considering the superposition of two pathways via high- and low oxidation state intermediate. The model is compared with experimental data measured in acidic media^4,15^. For the high oxidation state path (‘HOSP’), the same mechanism shall be assumed as in section “Thermal Iridium oxide” for the TO-model. Regarding the low oxidation state path (‘LOSP’), an Ir(III)-intermediate^4^ was proposed as well as a LOER-mechanism^6,15^. In terms of microkinetic network structure, the latter two mechanisms share the same characteristic of a re-oxidation reaction step directly following the unstable intermediate. Therefore, the same ($${C}_{diss}/EC)$$-structure holds for the two pathways if dissolution due to a lattice oxygen vacancy is assumed via a chemical step to $$I{r}^{3+}$$. In the following, the Ir(III)-path depicted in Fig. 5 is considered for the LOSP, keeping in mind its structural similarity to the LOER-mechanism.

Two types of active sites are considered, which contribute to the OER and dissolution either via high or low oxidation state intermediate path. Only the Temkin parameter and the dissolution rate constant are varied for the HOSP-model, compared to the model of the TO-surface, to account for a different morphology and a different ${IrO}_{2}$ lattice stability^46^, related to the thermal preparation process^43^. The ratio of the two sites is considered as a surface property and fitted together with the kinetic parameters of the Ir(III)-path. The increase in effective surface area, and thus the total number of active sites, is approximated by multiplying the number of active sites of the TO-surface by 11.7, a value found for the reduction factor of the anodic charge between 0.4 and 1.4 V after thermal treatment of a spin deposited Iridium oxide surface at 550 °C^60^. Temkin adsorption kinetics are considered for the hydroxyl adsorption step of the Ir(III)-path, to account for the effect of surface morphology and resulting heterogeneity of adsorption energy levels on the RS-surface.

#### Model discussion

The simulation results are shown in Fig. 6a and b. The RS-model shows good agreement to the experimentally observed OER activity and dissolution rates. The fact that a linear regression of the measured dissolution rates of the RS-surface does not cross the zero point of current density, is well captured by the RS-model through the strong dissolution via LOSP at low potentials. The model predicted dissolution rate approaches zero towards zero current density as expected for quasi-steady-state conditions on Iridium oxide. The inset in Fig. 6a visualises the shift of onset potential for the two pathways. The model predicted maximum of dissolution via LOSP is the result of the increasing re-oxidation kinetics relative to the saturating kinetics of Ir(III) formation via LOSP oxygen evolution. The model suggests that the high activity of the RS-surface at low current densities is due to the significant contribution of the LOSP, while at high current densities, the higher activity is increasingly extrinsic, related to a larger number of active sites. At high potentials, the Tafel slope of the LOSP is higher than that of the HOSP, since the saturation of the rds precursor of the LOSP starts at lower potentials compared to the HOSP. The superposition of mechanisms and the transition towards activity via HOSP can explain the experimentally observed, relatively high slope and its continuous bending (Fig. 6b). It seems likely that the model fit overestimates the Temkin parameter, as mass transport is not considered in the model, which can have a similar effect of curve bending. However, the observation that the Temkin parameter for the high oxidation state path in the RS-model is higher than that of the TO-model goes in line with an expected lower surface heterogeneity of the TO-surface, as a result of the thermal preparation step.

Figure 2 shows that high and low oxidation state pathways are characterised by constant and increasing selectivity, respectively. As a result, the superposition-model predicts an increase in selectivity followed by a constant region. The increase in selectivity at low current densities is explained by the direct competition of an electrochemical OER-step with a chemical dissolution step. The constant selectivity region is the result of the HOSP becoming dominant at high current densities. The experimental data in Fig. 2 show that the TO-surface incorporates a higher selectivity also at higher current densities, even though the model suggests that the same mechanistic path dominates in this region with both surfaces. This can be rationalised considering the effect of a more stable lattice of the TO-surface, due to the smaller number of surface imperfections^61^ and the stability of specific lattice orientations^46^. At the current density of 20 mA/cm^2^, the experimental data of the RS-surface might suggest the onset of a decrease in selectivity in a similar way as for example metallic and hydrous $Ir{O}_{x}$, which show a maximum in selectivity (Fig. 2). In this case, it may be hypothesised that the model of the RS-surface misses an important mechanistic feature in the high current density region. A kinetic explanation for such maximum in selectivity could be the onset of an additional, strongly potential dependent dissolution path. This dissolution path would need to have stronger potential dependence than the OER path, since the selectivity decreases with potential only when the dissolution slope is lower than that of the OER.

On the other hand, also the effect of mass transport can cause a decrease in selectivity when it hinders an increase in the reaction rate of the OER ‘reaction branch’ more than proportionally to that of the dissolution reaction. If assumed that the mechanistically relevant dissolution step does not require supply or removal of species such as $${H}^{+}$$ or $${H}_{2}O$$ via mass transport (e.g. $$Ir{O}_{2}$$–$$O$$$$\to Ir{O}_{3}$$), increasing mass transport limitations can be expected to decrease the selectivity. We suggest that experimental studies accessing high current densities under purposeful variation of mass transport related boundary conditions can provide further insights on this aspect and help in the discrimination of mechanistic explanations. Moreover, evaluation of the mechanistic proton reaction order of the RS-model and comparison to experiments can further validate or modify the derived mechanistic model. Supplementary Figs. 1 and 2 show that, due to the superposition of pathways, the RS-model predicts a distinct proton reaction order for both activity and stability. The reaction order does not show an extended region of potential independence, in contrast to that predicted by the TO-model. These observations provide a basis for targeted, model-based design of experiments for mechanistic discrimination.

Since the pathway via low oxidation state intermediate shows a significantly lower selectivity, considering stability it seems beneficial to avoid the OER via this path. It has been proposed that also hydrous $Ir{O}_{x}$ type surfaces undergo OER via low oxidation state intermediate^6,12^. Although the phenomena of oxygen evolution and catalyst dissolution appear highly coupled, the different behaviours of activity and stability of $TO$–$Ir{O}_{2}$ and RS–$Ir{O}_{2}$ indicate that morphology can have an important influence on the ratio of effective OER vs dissolution rate. This is supported by finding that $Ir{O}_{2}$(110), which is considered the thermodynamically most stable lattice plane termination^43,62,63^, shows significantly less surface roughening, even when polarised to higher current densities, than $Ir{O}_{2}$(100)^46^. Reduction of lattice imperfections such as grain boundaries can likely increase overall lattice stability^61^. Furthermore, the creation of nanoporous structure, as for example by dealloying Osmium from an $I{r}_{25}O{s}_{75}$ alloy, results in significantly enhanced OER/dissolution selectivity, possibly due to the effect of the concentration of dissolved species confined within the nanopores^64^. These insights should be considered in the search for more active and stable OER electrocatalyst materials.

## Conclusion

In this work, based on different quasi-steady-state and quasi-equilibrium modelling assumptions, it is analysed how the microkinetic network structure of an electrocatalytic cycle, in which a common intermediate causes catalyst dissolution, governs the interplay between electrocatalytic activity and stability. The potential dependence of the OER over dissolution selectivity is in particular determined by the types of the dissolution and reaction steps of the competing ‘reaction branches’ the common intermediate can undergo. The analysis indicates that dissolution via a chemical reaction step is associated with either a constant or increasing OER over dissolution selectivity. Characteristic reaction sequences, such as the re-oxidation step of the lattice defects within the lattice-participation OER mechanism or dimerisation reaction steps resulting in a quadratic reaction order, can be considered to microkinetically discriminate between mechanistic models. The derived concepts are applied to analyse the coupling of OER and catalyst dissolution on rutile and reactively sputtered Iridium oxide surfaces. Mathematical models are derived for the two surface types, which are well in line with activity and stability data. For rutile Iridium oxide surfaces, the experimentally observed activity and stability behaviour can be well described by a sequential adsorbate evolution path and the direct competition of a mononuclear and chemical rate-determining step with a chemical dissolution step. For the reactively sputtered Iridium oxide surface, experimentally observed characteristics are well captured by the additional assumption of a low oxidation state intermediate path, conceivably including lattice oxygen participation. High and low oxidation state pathways are suggested to govern the activity and stability behaviour at high and low current densities, respectively. The increase in OER over dissolution selectivity with potential at low current densities is comprehensible within the low oxidation state intermediate path, through the competition between an electrochemical re-oxidation and a chemical dissolution reaction step. It is suggested that, while at low potentials, the higher intrinsic activity and dissolution rate of the reactively sputtered surface is of strong significance, at higher potentials, the difference to the thermal Iridium oxide surface is increasingly extrinsic, related to a higher number of active sites.

## Supplementary information

Supplementary Information.
