# Supplementary material for: On the role of microkinetic network structure in the interplay between oxygen evolution reaction and catalyst dissolution
Source: Sci Rep. 2020 Aug 24;10:14140. doi: 10.1038/s41598-020-69723-3 (PMC7445268; doi:10.1038/s41598-020-69723-3)
Supplement: Supplementary file 1 — Supplementary Information. [file 41598_2020_69723_MOESM1_ESM.pdf]

Supplementary information

On the role of microkinetic network structure in the interplay between oxygen evolution reaction and catalyst dissolution

An Phuc Dam[1], Georgios Papakonstantinou[1], Kai Sundmacher[1,2]

[1]Max Planck Institute for Dynamics of Complex Technical Systems, Department Process Systems Engineering, Sandtorstr.1, D-39106 Magdeburg, Germany
[2]Otto-von-Guericke University Magdeburg, Department of Process Systems Engineering, Universitätsplatz 2, D-39106 Magdeburg, Germany

Derivation of the OER over dissolution selectivity

A schematic representation of the discussed generic reaction network structure is given in Figure 1. ϑ shall be defined as the ratio of redeposition $r_{diss,b}$ to dissolution rate $r_{diss,f}$:

$$\vartheta := \frac{r_{diss,b}}{r_{diss,f}}$$

The concentration of dissolved species in the electrolyte c_{sol} can then be calculated depending on ϑ:

$$r_{diss,b} = r_{diss,f} \cdot \vartheta$$

$$c_{sol} k_{diss,b} \exp\left(-z^{diss}(1-\beta)\, f\, E\right) = \Theta_{n_c}\, k_{diss,f} \exp\left(z^{diss} \beta\, f\, E\right)\ \vartheta$$

$$c_{sol} = \vartheta\, \Theta_{n_c} \frac{k_{diss,f}}{k_{diss,b}} \exp\left(z^{diss} f\, E\right)$$

Θ_{n_c} denotes the coverage corresponding to the common intermediate of OER and dissolution and $f = F/(R\,T)$. $z^{diss} = 1$ and $z^{diss} = 0$ is considered for the dissolution step of electrochemical and chemical type, respectively. Note that the standard reversible potential is lumped into the rate constants[1–3].

Case 1: Defining $\bar{\mathbf{k}}_{\mathbf{diss}}$ for the case of dissolution step in QE ($\dot{\mathbf{V}}$ very low, $\boldsymbol{\vartheta} = \mathbf{1}$):

While for batch experiments under equilibrium conditions the net dissolution rate is zero, for flow cell experiments a net dissolution rate is observed despite of QE-conditions since fresh electrolyte is introduced into the system. The dissolution rate can then be described by:

$$r_{diss} = \frac{\dot{V}\, c_{sol}}{A} = \vartheta\, \Theta_{n_c}\, \frac{\dot{V}}{A}\frac{k_{diss,f}}{k_{diss,b}} \exp\left(z^{diss} f\, E\right) = \Theta_{n_c}\, \bar{k}_{diss} \exp\left(z^{diss} f\, E\right)$$

With the definition for $\bar{k}_{diss}$:

$$\bar{k}_{diss}(\vartheta = 1) := \frac{\dot{V}}{A}\frac{k_{diss,f}}{k_{diss,b}} \qquad \text{SI (1)}$$

Case 2: Defining $\bar{\mathbf{k}}_{\mathbf{diss}}$ for the case of dissolution >> redeposition ($\dot{\mathbf{V}}$ very high, $\boldsymbol{\vartheta} = \mathbf{0}$):

For $\vartheta = 0$ the apparent rate constant shall be defined as $k_{diss}^{app} := \bar{k}_{diss} \neq \mathrm{fun}(\dot{V})$:

$$r_{diss} = \Theta_{n_c}\, k_{diss,f} \exp\left(z^{diss} \beta\, f\, E\right) = \Theta_{n_c}\, \bar{k}_{diss} \exp\left(z^{diss} \beta\, f\, E\right)$$

With the definition for $\bar{k}_{diss}$:

$$\bar{k}_{diss}(\vartheta = 0) := k_{diss,f} \qquad \text{SI (2)}$$

Merging the two cases ($\vartheta = 1$ and $\vartheta = 0$) in a compact expression

An apparent kinetic rate constant k_{diss}^{app} is then given with:

$$k_{diss}^{app} = \bar{k}_{diss} \exp\left(z^{diss} \beta (1 + \vartheta) f E\right) \qquad \text{SI (3)}$$

Applying the QE-assumption:

$$r_{1,f} = r_{1,b}$$
$$\Theta_0 \overrightarrow{k}_0 \exp(\beta f E) = \Theta_1\, \overleftarrow{k}_1 \exp(-(1-\beta) f E)$$

With symmetry factor $\beta = 0.5$:

$$\leftrightarrow \quad \Theta_1 = \Theta_0 K_1 \exp(fE)$$

With $K_i = \frac{k_{I,f}}{k_{I,b}}$. Analogously:

$$\Theta_2 = \Theta_1 K_2 \exp(fE)$$

Inserting Θ_1:

$$\Theta_2 = \Theta_0 K_2 K_1 \exp(2fE)$$

For Θ_i it can be stated:

$$\Theta_i = \Theta_0 \exp([1 + 2(i-1)\beta]\, f\, E) \prod_{j=1}^{i-1} K_j \qquad \text{SI (4)}$$

Using the definition of the stability-number

Similar to the dissolution step, for the rds the effective kinetic rate constant can be expressed in a compact way considering z^{rds}=1 for an electrochemical step and $z^{rds} = 0$ for a chemical step:

$$\bar{k}_{rds} = k_{rds} \exp\left(z^{rds}\beta fE\right)$$

The selectivity can be defined as the stability number S_{num} which is the ratio of OER rate over dissolution rate[4]:

$$S_{num} = \frac{R_{OER}}{R_{diss}} = \frac{\Theta_{rds}^{\nu} \cdot \bar{k}_{rds}}{\Theta_{n_c} \cdot k_{diss}^{app}} \qquad \text{SI (5)}$$

Here, ν is the reaction order of the rate-determining step and is determined by the number of (adjacent) reactant species that need to be formed such that the rds can occur.

Derivation of selectivity for a mono-nuclear rds ($\nu = 1$)

This case results in a more simple derivation since the coverage Θ_{n_c} that represents the coverage of the unstable intermediate can be cancelled out:

$$S_{num} = \frac{\Theta_{n_{rds}}\, \bar{k}_{rds}}{\Theta_{n_c}\, k_{diss}^{app}} \qquad \text{SI (6)}$$

Analogously to equation SI (4), Θ_{rds} can be correlated to Θ_{n_c} and inserted into equation SI (6):

$$S_{num} = \frac{\Theta_{n_c} \exp([1 + 2(n_{EC} - 1)\beta]\, f\, E)}{\Theta_{n_c}\, k_{diss}^{app}}\, \bar{k}_{rds} \prod_{j=n_c}^{n_{rds}-1} K_j \qquad \text{SI (7)}$$

$n_{EC} = n_{rds} - n_c$ here represents the number of electrochemical type steps between the common intermediate and the rds. The nominator of equation SI (7) represents OER activity and is analogous to classic theory for Tafel-slope analysis. The derivation of selectivity in the mononuclear case can be considered as the simpler case since the influence of the coverage simply, mathematically cancels out. As a next step, the type of the dissolution step shall be further defined using equation SI (3):

$$S_{num} = \frac{\exp([1 + 2(n_{EC} - 1)\beta]fE) \cdot\ k_{rds} \exp\left(z^{rds}\beta fE\right)}{\bar{k}_{diss} \exp(z^{diss}\beta(1 + \vartheta)fE)} \prod_{j=n_c}^{n_{rds}-1} K_j$$

z^{rds} and z^{diss} are one if the step is electrochemical and zero if the step is of chemical type. Finally gives for the case of mononuclear rds ($\nu = 1$):

$$S_{num} = \exp\left(\left[n_{EC} + \left(z^{rds} - z^{diss}(1 + \vartheta)\right)\beta\right]\, f\, E\right) \frac{k_{rds}}{\bar{k}_{diss}} \prod_{j=n_c}^{n_{rds}-1} K_j \qquad \text{SI (8)}$$

And the derivative with respect to potential:

$$\frac{dS_{num}}{dE} = \exp\left(\left[n_{EC} + \left(z^{rds} - z^{diss}\right)\beta\right]fE\right) \cdot \left[n_{EC} + \left(z^{rds} - z^{diss}(1 + \vartheta)\right)\beta\ \right]\ f\ \frac{k_{rds}}{\bar{k}_{diss}} \prod_{j=n_c}^{n_{rds}-1} K_j \qquad \text{SI (9)}$$

<u>Derivation of selectivity for a bi-nuclear rds ($\nu = 2$)</u>

Plugging in equation SI (4) for $\Theta_{n_{rds}}$ and Θ_{n_c} gives:

$$S_{num} = \frac{\Theta_0^2 \exp(2[1 + 2(n_{rds} - 1)\beta]fE) \prod_{j=1}^{n_{rds}-1} K_j\, \bar{k}_{rds}}{\Theta_0 \exp([1 + 2(n_c - 1)\beta]fE)\ \left(\prod_{j=1}^{n_c} K_j\right)\ k_{diss}^{app}} \qquad \text{SI (10)}$$

The closing condition can be expressed as:

$$1 = \sum_{m=0}^{n_{rds}} \Theta_m \qquad \text{SI (11)}$$

Insertion of equation SI (4) gives:

$$1 = \sum_{m=0}^{n_{rds}} \Theta_0 \exp([1 + 2(m - 1)\beta]fE) \prod_{j=1}^{m} K_j$$

$$\Theta_0 = \frac{1}{\sum_{m=0}^{n_{rds}} \exp([1 + 2(m - 1)\beta]fE) \prod_{j=1}^{m} K_j} \qquad \text{SI (12)}$$

Plugging equation SI (12) into equation SI (10) one then obtains for the selectivity:

$$S_{num} = \frac{\left(\prod_{j=1}^{n_{rds}-1} K_j\right)^2 \bar{k}_{rds}}{k_{diss}^{app} \left(\prod_{j=1}^{n_c} K_j\right) \left(\sum_{m=0}^{n_{rds}} \exp([2(n_c + m - 2n_{rds})\beta]\, f\, E) \prod_{j=1}^{m+1} K_j\right)}$$

Furthermore, by using the definitions $n_{EC} = n_{rds} - n_c$ and for the dissolution step equation SI (3):

$$S_{num} = \frac{\left(\prod_{j=1}^{n_{rds}-1} K_j\right)^2 k_{rds} \exp\left(z^{rds} f\, \beta E\right)}{\bar{k}_{diss} \exp(z^{diss}\beta(1 + \vartheta)fE) \left(\prod_{j=1}^{n_c} K_j\right) \left(\sum_{m=0}^{n_{rds}} \exp([2(m - n_{rds} - n_{EC})\beta]\, f\, E) \prod_{j=1}^{m+1} K_j\right)}$$

And finally:

$$S_{num} = \frac{\left(\prod_{j=1}^{n_{rds}-1} K_j\right)^2 k_{rds}}{k_{diss} \left(\prod_{j=1}^{n_c} K_j\right) \left(\sum_{m=0}^{n_{rds}} \exp([m - n_{rds} - n_{EC} + \beta\{z^{diss}(1 + \vartheta) - z^{rds}\}]f\, E) \prod_{j=1}^{m+1} K_j\right)} \qquad \text{SI (13)}$$

And for the derivative with respect to potential (Z denotes the term in brackets in equation SI (13)):

$$\frac{dS_{num}}{dE} = -\frac{\left(\prod_{j=1}^{n_{rds}-1} K_j\right)^2 k_{rds}}{\left(k_{diss}\left(\prod_{j=1}^{n_c} K_j\right)\left(\sum_{m=0}^{n_{rds}} \exp(Z\,f\,E) \prod_{j=1}^{m+1} K_j\right)\right)^2} \cdot \sum_{m=0}^{n_{rds}}\left(\exp(Z\,f\,E) \cdot \left[m - n_{rds} - n_{EC} + \beta\left\{z^{diss}(1+\vartheta) - z^{rds}\right\}\right] f \prod_{j=1}^{m+1} K_j\right) \qquad \text{SI (14)}$$

Non-decreasing selectivity for a mononuclear mechanism with a chemical dissolution step without QE-assumption

In this section the non-decreasing selectivity in case of a chemical type dissolution step shall be demonstrated without using the QE-assumption. The selectivity is expressed as:

$$S_{num} = \frac{r_{n_{rds}}}{r_{diss}} = \frac{r_{n_C}}{R_{diss}} = \frac{r_{n_c,f} - r_{n_c,b}}{R_{diss}} = \frac{\Theta_{n_c}\,\bar{k}_{f,n_c}}{\Theta_{n_c}\,k_{diss}^{app}}$$

An auxiliary variable M shall be introduced to describe the OER-step following the common intermediate:

$$M := \frac{r_{n_c,f}}{r_{n_c,b}}$$

Since a net oxidation reaction is considered it holds $1 < M < \infty$. The two extremal cases are firstly described by M→1 which represents the QE-case ($R_{n_c,f} \approx R_{n_c,b}$) and secondly the opposite case M→∞ of non-equilibrium ($R_{n_c,f} \gg R_{n_c,b}$). For the selectivity it then holds:

$$S_{num} = \frac{r_{n_c,f} - r_{n_c,b}}{r_{diss}} = \frac{r_{n_c,f}\left(1 - \frac{1}{M}\right)}{R_{diss}}$$

Considering the forward reaction as an oxidation reaction without loss of generality the selectivity can be expressed as:

$$S_{num} = \frac{\Theta_c\,\bar{k}_{f,n_c}}{\Theta_c\,k_{diss}^{app}} = \frac{k_{rds}\exp\left(z_{n_c}\beta f E\right)}{k_{diss}^{app}}\left(1 - \frac{1}{M}\right)$$

Where z_{n_c} (=0,1,2, …) is the number of electrons transferred in the competing OER reaction step. Furthermore, with the chemical reaction constant $k_{diss}^{app} \neq f(E)$:

$$\frac{dS_{num}}{dE} = \frac{1}{k_{diss}^{app}} k_{rds} \exp(z_{n_c}\beta fE) \cdot z_{n_c}\beta f \left(1 - \frac{1}{M}\right) \qquad \text{SI (15)}$$

Since $\left(1 - \frac{1}{M}\right) \geq 0$, the right hand side equation SI (15) is positive and therefore selectivity does not decrease with potential $\left(\frac{dS_{num}}{dE} \geq 0\right)$, when the common intermediate dissolves via chemical reaction step. Given an electrochemical reaction step in competition with dissolution $z_{n_c} > 0$ and thus selectivity increases with potential $\left(\frac{dS_{num}}{dE} > 0\right)$.

<u>Derivation selectivity for direct competition of rds with catalyst dissolution without QE-assumption</u>

In case of direct competition between rds and dissolution (cases 1-4 in Table 2) the selectivity describtion can be derived without the QE-assumption:

$$S_{num} = \frac{\Theta_{n_{rds}}\, \bar{k}_{rds}}{\Theta_{n_c}\, k_{diss}^{app}} = \frac{\Theta_{n_c}\, \bar{k}_{rds}}{\Theta_{n_c}\, k_{diss}^{app}} = \frac{\bar{k}_{rds}}{k_{diss}^{app}} = \frac{k_{rds} \exp(z^{rds}\beta fE)}{\bar{k}_{diss} \exp(z^{diss}\beta(1+\vartheta)fE)}$$

It follows:

$$S_{num} = \exp\left(\left[\left(z^{rds} - z^{diss}(1+\vartheta)\right)\beta\right] f\,E\right) \frac{k_{rds}}{\bar{k}_{diss}} \qquad \text{SI (16)}$$

Which is the same expression as equation SI (8), when $n_c = n_{rds}$, however here derived without using the QE-assumption.

Supplementary chemical reaction equations – Reactions and intermediates

Supplementary Table S1 – Possible reaction equations for the microkinetic network structure used for modelling the rutile IrO_2 surface (Figure 3c).

$$S + H_2O \rightleftharpoons S\text{-}OH + H^+ + e^- \qquad (R_3)$$

$$S\text{-}OH \rightleftharpoons S\text{-}O + H^+ + e^- \qquad (R_4)$$

Towards oxygen evolution		Towards dissolution	
$S\text{-}O + H_2O \rightleftharpoons SO\text{-}OH^- + H^+$	(R_5)	$IrO_2\text{-}O \rightleftharpoons IrO_3$	(R_6)
$SO\text{-}OH^- \rightleftharpoons S\text{-}O_2^- + H^+ + e^-$	(R_1)	$IrO_3 + H_2O \rightleftharpoons IrO_4^{2-} + 2H^+$	
$S\text{-}O_2^- \rightleftharpoons S + O_2 + e^-$	(R_2)		

Supplementary Table S2 – OER reaction intermediates as proposed in literature

Index i of Θ_i	OER via IrO_3-path[5,6], Figure 3b	Kobussen-path for OER[7], Figure 3c	OER via Ir(III)-path[5], Figure 5
0	IrO_2	$SO\text{-}OH^-$	$HIrO_2$
1	$IrO_2\text{-}OH^-$	$S\text{-}O_2^-$	IrO_2
2	IrO_2OH	S	$IrO_2\text{-}OH^-$
3	IrO_3	S-OH	IrO_2OH
4		S-O	

Model equations

TO-model and high oxidation state path of the RS-model (HOSP):

$$r_{3,f} = a_{H_2O}\Theta_2 k_{3,f} \exp(\beta f E)$$

$$r_{3,b} = a_{H^+}\Theta_3\, k_{3,b}(-(1-\beta) f E)$$

$$r_{4,f} = \Theta_3 k_{4,f} \exp(\beta f E)$$

$$r_{4,b} = a_{H^+}\Theta_4 k_{4,b}(-(1-\beta) f E)$$

$$r_{5,f} = k_{5f}\, a_{H_2O}\Theta_4$$

$$r_{5,b} = k_{5,b} a_{H^+} \Theta_0$$

$$r_{1,f} = \Theta_0 k_{4,f} \exp(\beta f E)$$

$$r_{1,b} = k_{1,b} a_{H^+} \Theta_1 (-(1-\beta) f E)$$

$$r_{2,f} = \Theta_1 k_{2,f} \exp(\beta f E)$$

$$r_{2,b} = a_{O_2} \Theta_2 k_{2,b} \exp(-(1-\beta) f E)$$

$$r_6 = \Theta_4 k_{6,b}$$

$$0 = (r_{2,f} - r_{2,b}) - (r_{3,f} - r_{3,b})$$

$$0 = (r_{3,f} - r_{3,b}) - (r_{4,f} - r_{4,b})$$

$$0 = (r_{4,f} - r_{4,b}) - (r_{5,f} - r_{5,b}) - r_6$$

$$1 = \sum_{i=0}^{4} \Theta_i$$

$$i_{HOSP} = \Gamma_\infty^{TO} F \, 4 \left(r_{2,f} - r_{2,b}\right)$$

<u>RS-surface (Ir(III)-path) model, low oxidation state path (LOSP):</u>

$$r_{2,f} = a_{H_2O} \Theta_1 k_{2,f}$$

$$r_{2,b} = a_{H^+} \Theta_3 \, k_{2,b}$$

$$r_{3,f} = \Theta_2 k_{3,f} \exp\left(\beta \left[fE - \frac{r_{tem}\Theta}{RT}\right]\right)$$

$$r_{3,b} = a_{H^+} \Theta_3 k_{3,b} \exp\left(-(1-\beta)\left[fE - \frac{r_{tem}\Theta}{RT}\right]\right)$$

$$r_{4,f} = k_{4f} \Theta_4^2$$

$$r_{4,b} = k_{4,b} a_{O_2} \Theta_0$$

$$r_{1,f} = \Theta_0 k_{1,f} \exp(\beta f E)$$

$$r_{1,b} = k_{1,b} a_{H^+} \Theta_1 (-(1-\beta) f E)$$

$$r_{5,f} = k_{5,f} \, \Theta_0 a_{H^+}^3$$

$$\Theta = \sum_{i=1}^{3} \Theta_i$$

$$0 = (r_{2,f} - r_{2,b}) - (r_{3,f} - r_{3,b})$$

$$0 = (r_{3,f} - r_{3,b}) - (r_{4,f} - r_{4,b})$$

$$0 = (r_{4,f} - r_{4,b}) - (r_{1,f} - r_{1,b}) - r_5$$

$$0 = (r_{1,f} - r_{1,b}) - (r_{2,f} - r_{2,b})$$

$$1 = \sum_{i=0}^{4} \Theta_i$$

$$i_{LOSP} = \Gamma_\infty^{RS}(1-\lambda) F\, 4\left(r_{4,f} - r_{4,b}\right)$$

$$i_{HOSP} = \Gamma_\infty^{RS}\, \lambda\, F\, 4\left(r_{2,f}^{HOSP} - r_{2,b}^{HOSP}\right)$$

$$i_{tot} = i_{LOSP} + i_{HOSP}$$

<u>Simulation parameter values</u>

Supplementary Table S3 - Kinetic rate constants for OER /dissolution via TO-model:

Parameter	Value [1/s]	Parameter	Value [1/s]
$\mathbf{k_{1,f}}$	1.00E+30	$k_{1,b}$	1.00E+10
$\mathbf{k_{2,f}}$	1.00E+30	$k_{2,b}$	1.00E+10
$\mathbf{k_{3,f}}$	8.07E-01	$k_{3,b}$	1.32E+19
$\mathbf{k_{4,f}}$	1.17E-08	$k_{4,b}$	1.64E+22
$\mathbf{k_{5,f}}$	1.54E+02	$k_{5,b}$	4.51E-04
$\mathbf{k_{6,f}}$	2.10E-03	r_{tem} [J/mol]	25650

Supplementary Table S4 - Kinetic rate constants for OER /dissolution via HOSP:

Parameter	Value [1/s]	Parameter	Value [1/s]
$\mathbf{k_{1,f}}$	1.00E+30	$k_{1,b}$	1.00E+10
$\mathbf{k_{2,f}}$	1.00E+30	$k_{2,b}$	1.00E+10
$\mathbf{k_{3,f}}$	8.07E-01	$k_{3,b}$	1.32E+19
$\mathbf{k_{4,f}}$	1.17E-08	$k_{4,b}$	1.64E+22
$\mathbf{k_{5,f}}$	1.54E+02	$k_{5,b}$	4.51E-04
$\mathbf{k_{6,f}}$	3.80E-03	r_{tem} [J/mol]	89620

Supplementary Table S5 - Kinetic constants for OER-dissolution mechanism via LOSP:

Parameter	Value [1/s]	Parameter	Value [1/s]
$\mathbf{k_{1,f}}$	1.61E-09	$k_{1,b}$	1.01E+06
$\mathbf{k_{2,f}}$	2.73E+11	$k_{2,b}$	2.65E+07
$\mathbf{k_{3,f}}$	1.18E+04	$k_{3,b}$	1.58E+30
$\mathbf{k_{4,f}}$	1.23E+03	$k_{4,b}$	2.48E-05
$\mathbf{k_{5,f}}$	2.12E+02	r_{tem}	2.08E+04

Supplementary Table S6 - Other model parameters

Symbol	Unit	Value	Notes
$\mathbf{\Gamma_\infty^{TO}}$	mol/m^2	9.58E-06	Number of active sites per unit area for the TO-surface. Calculated from voltammetric charge[4]
$\mathbf{\Gamma_\infty^{RS}}$	mol/m^2	1.12E-04	Ratio of number of active sites before and after thermal treatment is approximated using the factor 11.7[8]
λ	[-]	0.88	Fraction of HOSP active sites compared to all active sites
$\boldsymbol{\beta}$	[-]	0.5	Symmetry factor
T	[K]	298	Temperature
$\mathbf{a_{H^+}}$	[mol/L]	0.1	Proton activity

Supplementary Table S7 - Other symbols

Symbol	Unit	Description
F	[A s/mol]	Faraday constant
$\mathbf{\Theta_i}$	[-]	Coverage of intermediate i
$\mathbf{i}$	[A/m^2]	Current density
$\mathbf{a_{O_2}}$	[-]	Oxygen activity in electrolyte solution (lumped into kinetic constant)
$\mathbf{a_{H_2O}}$	[-]	Water activity in electrolyte solution (lumped into kinetic constant)
$\mathbf{R}$	[J/(mol · K)]	Universal Gas Constant
$\mathbf{r_i}$	[1 / s]	Coverage reaction rate of reaction i

Calculation of mechanistic proton reaction order

The developed models were used to calculate the mechanistic proton reaction orders by applying the following equation:

$$m = \frac{\ln\left(\frac{R_x(E)}{R_y(E)}\right)}{\ln\left(\frac{a_{H,x}}{a_{H,y}}\right)}$$

For discussing OER-activity, R is the oxygen evolution reaction rate and for discussing stability R is the dissolution rate. x and y represent different pH values as given in the legend of Supplementary Figure 1-3. The potential E vs SHE is kept constant for evaluation of the mechanistic reaction order.

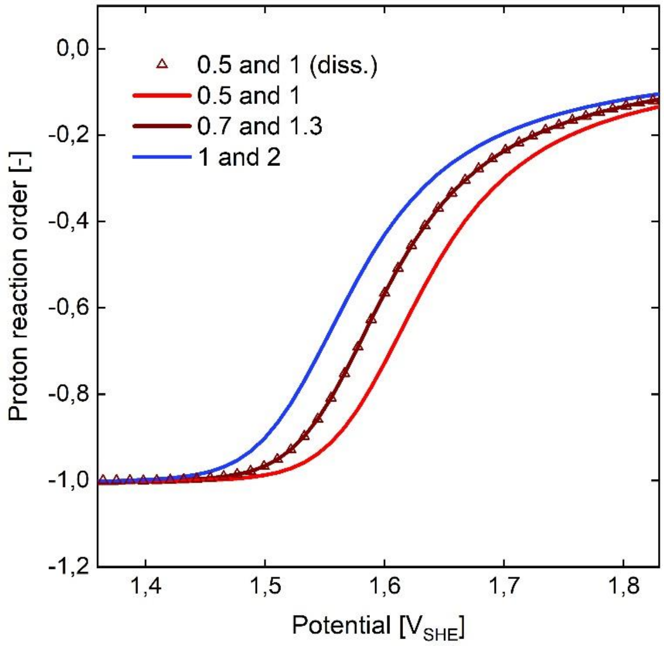

Supplementary Figure 1 – Model predicted reaction order the TO-model, evaluated for different pH in acidic region as given by the numbers. The rds does not contribute to an additional a_H dependence. The value of -1 is due to the electrochemical formation of adsorbed O including deprotonation. Towards theoretical limiting current conditions, the proton reaction order approaches zero. The proton reaction order of dissolution is predicted equal to that of OER reaction rate.

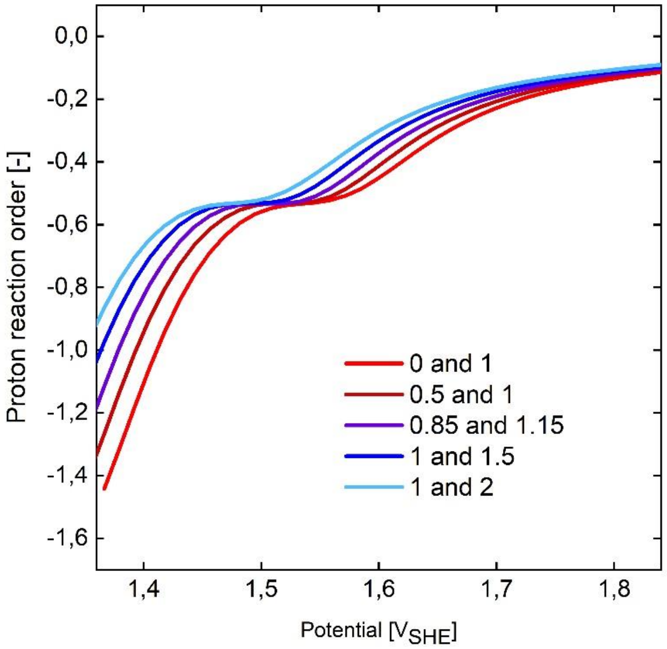

Supplementary Figure 2 – Predicted proton reaction order of OER activity by the RS-model, evaluated for different pH in acidic region as given by the numbers. The superposition of the two pathways results in a distinct, potential dependent reaction order behaviour. At low potentials, activity and therefore also the proton reaction order is dominated by the LOSP. The OH adsorption step in QE and a following bi-nuclear rds results in a reaction order of -2. Due to saturation of the adsorbed OH species, OER via LOSP saturates and the reaction order of the LOSP approaches zero. The transition between 1.5 and 1.6 V_{SHE} is explained by the superposition with the HOSP, which is still at a reaction order of ca. -1 at 1.5 V_{SHE}. Above 1.5 V_{SHE} the HOSP also starts to saturate with coverage of rds precursor.

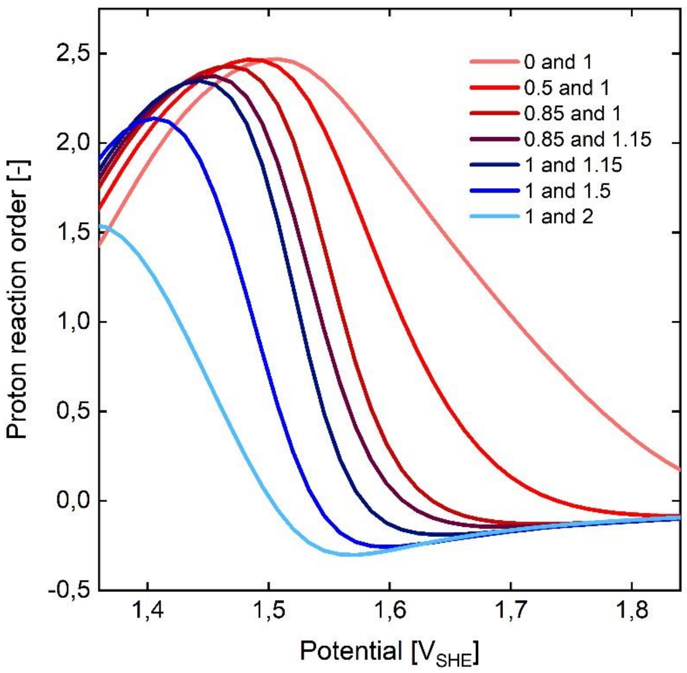

Supplementary Figure 3 – Mechanistic reaction order of Iridium dissolution predicted by the RS-model, evaluated for different pH in acidic region as given by the numbers. The apparent reaction order for the LOSP is a result of the interplay of the different reaction steps and their proton activity dependences. As explained in Supplementary Figure 2, the reaction order of the LOSP is -2 and similar is the dependence of the formation rate of Ir(III), which is considered prone to dissolution. On the other hand, the dissolution step demands the supply of 3 protons following the reaction equations[5] for the Ir(III) dissolution: $\mathrm{HIrO_2} + 3\mathrm{H^+} \rightarrow \mathrm{Ir^{3+}} + 2\ \mathrm{H_2O}$. Therefore, this dissolution reaction step incorporates a reaction order of +3. Since the backward reaction rate of the oxidation reaction from adsorbed OH to Ir(III) is suggested by the model fit to be negligibly small, this reaction step does not contribute to an additional effect. The observed maximum proton dependence is related to the maximum of Ir(III) coverage and dissolution via Ir(III), which is shown in Fig 6a. The HOSP contributes with a reaction order of -1, which is especially seen in the evaluation of higher pH. Similar to the reaction order for OER activity, the dissolution proton reaction order approaches zero towards limiting current conditions.

Supplementary Information References

1. Bockris, J. O.'M., Reddy, A. K. N. & Gamboa-Aldeco, M. E. *Modern electrochemistry. Fundamentals of Electrodics (Second Edition).* 2nd ed. (Plenum Press, New York, 1998-2000).
2. Gileadi, E. *Electrode kinetics for chemists, chemical engineers, and materials scientists* (VCH, New York, 1993).
3. Lefebvre, M. C. *Establishing the link between multistep electrochemical reaction mechanisms and experimental Tafel slopes* (1999).
4. Geiger, S. *et al.* The stability number as a metric for electrocatalyst stability benchmarking. *Nat. Catal.* **1,** 508–515 (2018).
5. Kasian, O., Grote, J.-P., Geiger, S., Cherevko, S. & Mayrhofer, K. J. J. The common intermediates of oxygen evolution and dissolution reactions during water electrolysis on Iridium. *Angew. Chem.* **57,** 2488–2491 (2018).
6. Kötz, R., Neff, H. & Stucki, S. Anodic Iridium oxide films. XPS-Studies of oxidation state changes and O_2-evolution. *J. Electrochem. Soc.* **131,** 72 (1984).
7. Kobussen, A.G.C. & Broers, G.H.J. The oxygen evolution on $La_{0.5}Ba_{0.5}CoO_3$. *J. Electroanal. Chem. Interf. Electrochem.* **126,** 221–240 (1981).
8. Reier, T. *et al.* Electrocatalytic Oxygen evolution on Iridium oxide: Uncovering catalyst-substrate interactions and active Iridium oxide species. *J. Electrochem. Soc.* **161,** F876-F882 (2014).
